# Sputum cell counts to manage prednisone-dependent asthma: effects on FEV_1_ and eosinophilic exacerbations

**DOI:** 10.1186/s13223-017-0190-0

**Published:** 2017-04-04

**Authors:** Afia Aziz-Ur-Rehman, Angira Dasgupta, Melanie Kjarsgaard, Frederick E. Hargreave, Parameswaran Nair

**Affiliations:** 1 Department of Medicine, McMaster University and St Joseph’s Healthcare Hamilton, Hamilton, ON Canada; 2 Firestone Institute for Respiratory Health, St. Joseph’s Healthcare, 50 Charlton Avenue East, Hamilton, ON L8N 4A6 Canada

**Keywords:** Severe asthma, Prednisone, Sputum cell counts, Eosinophils, FEV1, Exacerbations

## Abstract

**Background:**

Prednisone dependence in asthma is usually described based on clinical and spirometric characteristics. It is generally believed that these patients have frequent exacerbations and lose lung function rapidly because of uncontrolled airway eosinophilia.

**Objectives:**

The objectives of this study are to report the effect on asthma exacerbations and the change in lung function over time in prednisone-dependent asthma when severe asthma is managed using a protocol that aims to maintain normal sputum cell counts.

**Methods:**

A retrospective survey of patients prospectively assessed in a university tertiary care asthma clinic.

**Results:**

52 patients (30 males, mean age 51 years, 64% non-atopic) were followed for a median period of 5.4 years (min–max: 0.2–35.2). Monitoring with the aim of keeping sputum eosinophils below 3% resulted in higher doses of corticosteroids (median daily dose of prednisone was 10 mg and for inhaled corticosteroids was 1500 μg of fluticasone equivalent) than at baseline and this was associated with predictable adverse effects. Despite the disease severity, 10 patients (19%) did not require LABA for symptom control. Most importantly, over the period of follow-up, there were only 0.3 eosinophilic exacerbations/patient/year. Overall, there was an increase in FEV1 over the period of follow-up (mean +84.6 ml/year) rather than an expected decline.

**Conclusions:**

Monitoring of eosinophils in sputum enables to maintain symptom control and preserve FEV1 in patients with severe prednisone-dependent asthma.

## Background

Asthma management guided by sputum cell counts has been shown to reduce eosinophilic exacerbations [1, 2] and is cost-effective [3]. This is particularly true for patients with moderate to severe asthma as most patients with mild asthma may not require a biomarker-guided treatment strategy [4]. However, it is not known if patients with the severest forms of asthma i.e. those that require daily prednisone would also benefit from a sputum-based management strategy. It is generally believed that these patients have frequent exacerbations, particularly those with persistent sputum eosinophilia [5] and that they lose lung function over time with each exacerbation [6]. These patients, although fortunately infrequent, consume the largest health care resources for asthma care [7]. They often have significant adverse effects from their doses of corticosteroids [8] and these are the patients who may benefit most with the advent of biologics that target the Th2 cytokine pathways [9].

The recent experience from the British Thoracic Society Severe Asthma program suggest that the clinical outcomes of patients with severe asthma are better if they are managed in specialized asthma centres than in general clinics [10]. A severe asthma clinic was set up at the Firestone clinic at St Joseph’s Healthcare in Hamilton, ON in the early 1970s where patients were looked after by a respiratory physician (FEH) who was supported by a research staff of two technologists and one clinical trainee who was often a respiratory physician. The two unique features of this clinic were the introduction of quantitative cell counts in sputum to adjust initial treatment requirements and secondly (and more importantly) accessibility to these measurements within 72 h of any worsening of asthma symptoms. The main objectives of this manuscript are to describe the effects of this strategy on FEV_1_ and on exacerbations in patients with prednisone-dependent asthma who were referred to this clinic.

## Study design and methods

This was a retrospective descriptive chart review of patients with a physician-confirmed diagnosis of asthma (defined as episodic wheeze, chest tightness or shortness of breath and confirmed variable airflow obstruction of at least 12% and 200 ml improvement in FEV1 after inhaling 200 mcg of salbutamol or a PC20 methacholine of <8 mg/ml), and who were on a maintenance dose of at least 5 mg of prednisone daily for at least 6 months prior to the initial consultation, who were referred to a severe asthma clinic at the Firestone Institute in Hamilton, Ontario, between 1973 and 2008. Basic clinical and demographic data were documented. Pre-and post-bronchodilator reversibility were recorded according to the American Thoracic Society standards [11]. Airway responsiveness to methacholine was assessed by the tidal breathing method of Cockcroft et al. [12] if the FEV_1_ was >65% of predicted. Symptoms of cough, wheeze, chest tightness, dyspnea and sputum production were documented on a 7-point Likert scale (1 being worst and 7 being best). Tools to assess “asthma control” and “asthma-specific quality of life” were not available when the first patients were recruited into this program. Sputum was induced and processed according to the methods described by Pizzichini et al. [13].

Asthma was managed according to the protocol described by Jayaram et al. [1]. Briefly, the dose of inhaled corticosteroids or prednisone was increased to maintain sputum eosinophils less than 3% (or until free eosinophil granules were no longer present). If sputum total cell count was greater than 15 × 10^6^/g and neutrophils greater than 65%, the patients were treated with a broad spectrum antibiotic (zithromycin or amoxicillin + clavulanic acid for 5–7 days). Most importantly, the dose of steroid was not increased. Long-acting bronchodilators (salmeterol or formoterol) were added to the inhaled steroids only after the bronchitic component was controlled and the patient continued to have shortness of breath or wheezing that required more than 2–4 puffs of short-acting bronchodilators daily. They were not added if spirometry did not show any worsening of airflow obstruction or if PC20 methacholine was greater than 8 mg/ml or had not worsened by more than one doubling dose. If the sputum eosinophil % was less than 1%, the dose of corticosteroids was reduced. Sputum was always rechecked with 6–8 weeks of any treatment change. Once the maintenance dose of steroid was identified, patients were left on this dose indefinitely and seen in follow-up on average twice a year at which time spirometry, sputum and blood counts, clinical asthma control and adverse effects of therapy were assessed by self-reported history. Methacholine airway responsiveness was also reassessed if patients reported an increase need for short-acting bronchodilators and the sputum cell counts were normal and if it was felt safe to perform the test (usually FEV_1_ > 65% predicted). Adherence to prescribed medications was continuously assessed by checking the pharmacy records every year.

If patients experienced any worsening of symptoms (increase in chest tightness or wheezing requiring at least four puffs of salbutamol daily or at night, increase in sputum production or change in colour to dark yellow or green) they were instructed to call our research office. Patients were brought to the clinic within 72 h for a clinical assessment, spirometry, and collection of either spontaneously expectorated or induced sputum. They were phoned back the same evening or the next morning with instructions to change their medication dosages. If patients had seen their family doctor and had received either antibiotics or prednisone without being seen at our clinic, this information was documented in the clinic chart. All the demographic and clinical information was meticulously extracted by a research assistant (AAR) and verified by a research technologist (MK) after obtaining approval from the Hospital Research Ethics Board.

### Statistical analysis

Baseline demographic and clinical data were summarized using descriptive statistics. The rates of change of FEV_1_ (ml/year) were analyzed by multilevel linear regression using three time points (at baseline, time when sputum quantitative assay became normal and at the most recent assessment) for each gender and smoking status separately. As a first step, individual FEV_1_s were regressed against time to find rates of change (ml/year) for each patient. In the second step of multilevel linear regression, the rates of change (ml/year) for each patient (dependent variable) were regressed with age and height as the independent variables. The final rate of change of FEV_1_ in a specific group e.g. males, females, smokers and nonsmokers was computed using the mean age and height of the respective groups. The analyses were carried out using SPSS (version 16). Since we did not have a comparison group of patients with milder asthma or patients with severe asthma who were not monitored using sputum cell counts, we plotted the rates of decline of our cohort against the data published by Ulrik et al. [14] for patients with mild asthma. Paired data were compared by Student’s *t* test. P-value was considered significant if <0.05.

## Results

The study included 52 (30 males and 22 females) patients. The baseline characteristics of all patients are tabulated in Table 1. The median time of follow up of all patients was 5.42 years (minimum 0.15, maximum 35.26).

**Table 1:** Baseline characteristics (n = 52)

Age, years (mean, SD)	51 (11)
Male (n)	30
Smoker (n)	28
Atopy (n, %)	19 (36%)
Chronic rhinosinusitis (n, %)	23 (45%)
Aspirin sensitivity (n, %)	9 (18%)
Age of onset of symptoms, years (median, min–max)	20 (9–45)
Years on prednisone prior to initial assessment (mean, SD)	7.2 (6.6)
Number of courses of prednisone over past 2 years/patient/year (mean, SD)	1.8 (1.2)
Height, cm (mean, SD)	168.2 (10.2)
Weight, kg (mean, SD)	80.8 (14.4)
Serum IgE, KIU/l (mean, SD)	86 (18)
Blood eosinophil, ×10^3^/l (mean, SD)	0.4 (0.5)
ICS, µg (median)	1500
LABA (n)	22
LTRA (n)	14

### Effect on sputum cell counts

**Fig. 1:**
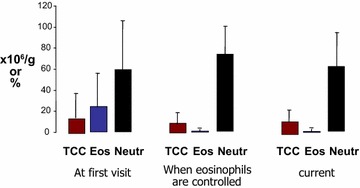
Sputum cell counts at first visit, when eosinophils are normalized, and current

Sputum eosinophil counts were normalized in all patients within a median period of 5 months (Table 2). This was associated with a trivial (but statistically significant) increase in sputum neutrophil % (Table 2; Fig. 1). The cell counts remained stable for the rest of the follow-up period.

**Table 2:** Sputum, blood counts and spirometry (mean, SD) values

	At initial visit	When sputum was normal	Current
Sputum			
Total cell count, ×10^6^/g	16 (24)	9 (11)	12 (8)
Eosinophil, %	22 (18)	1 (4)	2.4 (4.2)
Neutrophil, %	60 (49)	72 (28)	64 (20)
Blood			
Eosinophil count, ×10^3^/l	0.4 (0.5)	0.1 (0.2)	0.2 (0.3)
Eosinophil %	6 (8)	4 (8)	4 (6)
Spirometry			
FEV_1_, L	2.3 (0.8)	2.5 (0.8)	2.2 (0.8)
FEV_1_, %	70.7 (20.1)	76.9 (18.2)	69.4 (18.1)
VC, L	3.6 (1.1)	3.6 (0.9)	3.4 (1.1)
VC, %	88.7 (16.9)	90.9 (12.9)	84.4 (22.4)
FEV_1_/VC, %	63 (14)	65 (13)	64 (14)

### Effect on FEV_1_

The rate of change of FEV_1_ from baseline value to the time point when sputum quantitative assay became normal was 1201.24 ml/year (95% CI 199.31 to 2202.7 ml/year) while the rate of change (decline) from the time sputum was normal to the time when the patient was last seen was a modest −14.9 (95% CI 53.4 to −83.2) ml/year. The overall (baseline to when last seen) rate of change of FEV_1_ was 84.63 (95% CI −44.6 to 213.8) ml/year. The corresponding values in males were 970.53 (95% CI 178.5 to 1762.4) ml/year, −28.36 (95% CI −18.1 to −38.6) ml/year and the overall rate of change was 113.99 (95% CI 70.6 to 157.4) ml/year and for females were 1515.85 (95% CI −701.1 to 3732.7) ml/year, 3.44 (95% CI −115.7 to 122.6) ml/year and the overall rate of change was 44.59 (95% CI −95.6 to 184.8) ml/year. There were however no statistically significant difference between males and females in their rates of change of FEV_1_. The rates of change of FEV_1_ for male smokers were : from baseline to when sputum was normal 1433.13 (95% CI 199.3 to 2666.9) ml/year, from when sputum was normal to when last seen 6.09 (95% CI −113.1 to 125.3) ml/year and overall 227.31 (95% CI −91.9 to 546.5) ml/year and for females smokers were: from baseline to when sputum was normal 663.8 (95% CI −332.4 to 1660.0) ml/year, from when sputum was normal to when last seen 21.54 (95% CI −310.1 to 353.1) ml/year and overall 82.58 (95% CI −306.6 to 471.8) ml/year. The corresponding values for male nonsmokers were: 171.48 (95% CI −78.0 to 420.9) ml/year, −87.86 (95% CI −218.7 to 43.02) ml/year and −81.73 (95% CI −190.5 to 27.1) ml/year and for female nonsmokers were: 2105.74 (95% CI −1801.4 to 6012.9) ml/year, −9.09 (95% CI −35.3 to 17.2) ml/year and 18.28 (95% CI −11.4 to 47.9) ml/year. There were no statistical differences in rates of change of FEV_1_ between the genders when smokers and nonsmokers were analysed separately.

### Effect on exacerbations

Over the 2 years prior to attending our clinic, the patients had reported an average of 1.9 exacerbations/patient/year that had responded to prednisone. Since sputum was not examined during these exacerbations, we cannot confirm that these were eosinophilic, but we assume they were as patients reported improvement in their asthma symptoms within 48–72 h of therapy. This was reduced to 0.3 eosinophilic exacerbations/patient/year over the course of the follow-up period. The average time of resolution of individual exacerbations was 4 days. We did not have accurate records of “non-eosinophilic” or “neutrophilic” exacerbations before their initial visit to our clinic. During the course of the follow-up, the patients had 1.2 neutrophilic exacerbations/patient/year that were treated with antibiotics.

### Effects related to corticosteroids

The median duration of the steroid optimization phase was 5 months (min–max 1–7 months). During this period, the median daily dose of prednisone was 10 mg (minimum 5 mg, maximum 35 mg) and the dose of inhaled corticosteroid was 1500 mcg of fluticasone equivalent. This dose was maintained for the duration of the follow-up period. Five patients required the dose of prednisone to be increased after the maintenance dose was established. Corticosteroids caused predictable adverse effects (Table 3) that were appropriately managed.

**Table 3:** Co-morbidities and adverse effects of prednisone

	Prevalence (%)
Co-morbidities	
GERD	70
Sinusitis	65
Recurrent bronchitis	58
Polyps	45
BMI > 30	44
NSAID sensitivity	28
Neurosis	27
Adverse effects	
Osteopenia	72
Hypertension	60
Cataract	42
Skin bruising	35
Diabetes	16
Glaucoma	14

### Effects related to LABA

At initial assessment, 22 patients were on LABA (15 on salmeterol, 7 on formoterol). Over the course of the follow-up period, 20 patients were also commenced on LABA. The median period to commencement of LABA was 2 years (minimum 2 months, maximum 4 years). 10 patients have not required LABA for symptom control as their asthma severity (and airflow obstruction) was largely driven by steroid-responsive luminal eosinophilic inflammation rather than by bronchodilator-responsive smooth muscle dysfunction.

## Discussion

This retrospective study illustrates three important concepts. Firstly, when available, incorporation of timely measurements of sputum quantitative cytometry and airway hyperresponsiveness into routine clinical practice is feasible and effective in the management of severe prednisone-dependent patients with asthma. Secondly, this strategy can reduce exacerbations and preserve lung function albeit at the cost of adverse effects of glucocorticoids. Thirdly, recognition of the component of asthma that leads to severity can help to rationalize the inappropriate use of long-acting bronchodilators that are associated with asthma morbidity.

**Fig. 2:**
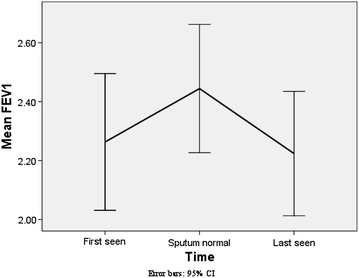
Mean FEV1 (with 95% CI) at three time points (when first seen, when sputum became normal, when last seen). *Asterisk* rates of decline are for the average age and height for the respective group

To our knowledge, this is the first study that reports the preservation of lung function in patients with severe asthma. Not only did we not observe the expected decline in FEV_1_ over time that has been reported in patients with “eosinophilic severe asthma” [5, 15], but there was a modest improvement over the period of observation suggesting that the current symptom-based guideline therapies underestimate the control of airway inflammation. We analysed longitudinal data using three time points i.e. when first seen, when sputum became normal and when a patient was last seen. The rate of change of FEV_1_ was positive when the rate was computed from the baseline value to the point when the airway inflammation (or the sputum quantitative assay) became normal (Fig. 2), whereas the rate of change of FEV_1_ thereafter, from the time sputum was normal to the time when the patient was last seen was a modest −14 ml/year which is clearly lesser than that reported in previous longitudinal asthma studies [6, 15–18]. The overall improvement in FEV_1_ in this study is possibly driven by the fact that most patients had an improvement in lung function when sputum became normal after intensive anti-inflammatory therapy and that too within a short span of time resulting in high rates of improvement in FEV_1_ with time. Comparison with a matched group of patients at the Firestone clinic with similar severity of asthma who were managed based on symptoms only would have certainly added strength to the study. Unfortunately, such data were not available to us. However, it is extremely unlikely that treatment based on symptoms alone would give results similar to treatment using a sputum strategy [1].

**Fig. 3:**
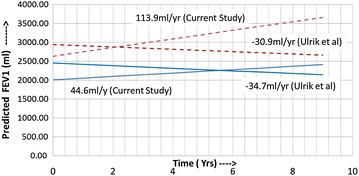
Comparison of predicted (calculated) FEV1 vs time in years (Current Study vs Ulrik et al.). *Dotted lines* males, *solid line* females; predicted rates of decline and FEV1 for both studies are for the mean age (males 52.4 years, females 49.8 years) and mean height (males 173.2 cm, females 161.4 cm) of the current study population. Equations for current study: FEV1 at time t for males = (2.6795 − 0.03808*AGE +0.01124*HT) +(1.05455 − 0.00646*AGE −0.00348*HT)t; FEV1 at time t for females = (−4.27125 − 0.0253*AGE +0.04674*HT) + (−2.71234 − 0.00336*AGE +0.01812*HT)t. Equations for Ulrik et al.: FEV1 at time t for males = (−469 − 35.2*AGE +32.0*HT) − (−107 − 0.79*AGE +0.6*HT + 1.7)t; FEV1 at time t for females = (−410 − 27.6*AGE +21.2*HT) − (−107 − 0.79*AGE +0.6*HT + 3)t

**Fig. 4:**
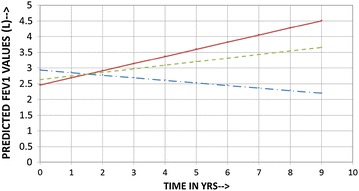
Predicted (calculated) FEV1 vs time in years for males (Baseline to last seen) showing no statistical difference between smokers and nonsmokers; *dashed line* all patients, *solid line* smokers, *dash* and *dot line* nonsmokers. Predicted rates of decline and FEV1 are for the mean age and mean height for the respective group of the current study population

**Fig. 5:**
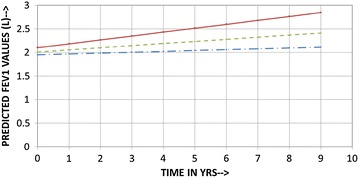
Predicted (calculated) FEV1 vs time in years in females (Baseline to last seen) showing no statistical difference between smokers and nonsmokers; *dashed line* all patients, *solid line* smokers, *dash* and *dot line* nonsmokers. Predicted rates of decline and FEV1 are for the mean age and mean height for the respective group of the current study population

Figure 2 illustrates a comparison of rates of change of FEV_1_ between this study and that by Ulrik et al. We fully realize that these two studies are not entirely comparable given the differences such as patient population, management strategies, sampling strategies etc. [14]. However, most longitudinal asthma studies are population based observational reports. We selected the Ulrick study as this is one of the larger longitudinal asthma studies and employed a similar statistical analysis method to what we performed. Various factors such as gender, smoking, age of onset, atopy, sputum eosinophilia, presence of mucous hyper secretion and use of inhaled corticosteroids have been implicated as affecting the rate of change in FEV_1_ in asthmatics. However, methodological issues have led to wide variations in these observations resulting in a lot of heterogeneity in the reported rates of decline in FEV_1_. Rates of decrease of FEV_1_ have varied from 25.7 ml/year in severe asthma [15], 31.5 ml/year in asthmatic patients with frequent exacerbations vs 14.6 ml/year in those with infrequent exacerbations [6], 26.6 ml/year in occupational asthmatics who were exposed to low-molecular-weight sensitizers at work [16], 28.4–39.7 ml/year in adult nonsmoker asthmatic patients in the Busselton cohort [17], to 16.1–21.5 ml/year in asthmatic patients receiving inhaled corticosteroids [18]. In the current study, the rate of decline from when sputum was normal to when last seen is similar to the decline rate reported in asthmatics with infrequent exacerbations [6]. Interestingly, in our study there were no statistical differences in rates of change of FEV_1_ either between the genders (Fig. 3) or when smokers and nonsmokers were analysed (Figs. 4, 5) separately. Regardless of the fact that it may not be apt to compare studies with dissimilar populations and methodologies, we were able to demonstrate, perhaps for the first time, a strategy that did not use biologics that could preserve lung function even when asthma is severe. Only the eosinophilic inflammatory component could be effectively targeted with corticosteroids. We did not have any effective or specific strategy (other than antibiotics) to treat neutrophilic bronchitis. We speculate that lung function could perhaps have been better preserved had broader strategies effective against non-T2 type inflammation as well been available for clinical use.

A second important point that we would like to highlight in this report is that patients could be on high doses of corticosteroids and not necessarily require long-acting beta-agonists if the severity is driven by luminal inflammation rather than by smooth muscle dysfunction. This is not often appreciated in clinical practice nor is it emphasized in the guidelines. In our cohort, the use of long-acting beta-agonist could be delayed or withheld in a small proportion of severe asthmatics in whom there is the highest concern for their adverse effects [19]. This also questions the veracity of recommendations to consider anti-eosinophil biologics (such as mepolizumab or reslizumab) as steroid-sparing therapy only after adding long-acting bronchodilators [20] when long-acting beta-agonists do not have any proven anti-eosinophil activities [21]. It is reasonable to consider biologics in patients whose disease are truly driven by eosinophils (as identified by persistent sputum eosinophils and blood eosinophils) and who have adverse effects from high doses of corticosteroids independent of their need for long-acting bronchodilators.

The major limitation of our study is the retrospective nature of data collection that spans over 25 years, the absence of a comparator group, and the lack of precision in the definition of exacerbations prior to referral to our clinic. It is also plausible that some of the exacerbations may not have been reported to us during the period of follow up. We did not use any sophisticated method to assess compliance other than self reports and pharmacy logs.

## Conclusion

In conclusion, we recommend monitoring of eosinophils in sputum in patients with severe prednisone-dependent asthma as this strategy enables to maintain symptom control, reduce exacerbations and preserve FEV_1_ in these patients. The test is not intrusive and is acceptable to most patients [22]. This study was performed before anti-IL5 monoclonal antibodies were available for clinical use but the results demonstrate that most patients would not require them for improving asthma control but they would be useful to avoid the adverse effects of corticosteroids. Our experience also provides ‘proof-of-principle’ that ‘remission’ [23] can be achieved even in severe asthma by judicious use of currently available therapy, albeit at the price of adverse effects of therapy.

## Abbreviations

FEV1: forced expiratory volume in the first second
PC20: provocative concentration to cause a 20% drop in FEV1
LABA: long-acting beta-agonist
ICS: inhaled corticosteroid
